# Bayesian phylodynamic analysis reveals the evolutionary history and the dispersal patterns of citrus tristeza virus in China based on the *p25* gene

**DOI:** 10.1186/s12985-023-02190-0

**Published:** 2023-10-03

**Authors:** Changning Wang, Chaoyun Chen, Yiqun Chen, Ke Zhong, Long Yi

**Affiliations:** 1 College of Life Sciences, Gannan Normal University, Ganzhou, 341000 China; 2 National Navel Orange Engineering Research Center, Ganzhou, 341000 China

**Keywords:** Citrus tristeza virus, Substitution rate, Phylogeography, Dispersal patterns

## Abstract

**Background:**

Citrus tristeza virus (CTV) is one of the most serious threats to the citrus industry, and is present in both wild and cultivated citrus. The origin and dispersal patterns of CTV is still poorly understood in China.

**Methods:**

In this study, 524 CTV suspected citrus samples from China were collected, including 354 cultivated citrus samples and 174 wild citrus samples. Finally, 126 CTV coat protein sequences were obtained with time-stamped from 10 citrus origins in China. Bayesian phylodynamic inference were performed for CTV origin and dispersal patterns study in China.

**Result:**

We found that CTV was mainly distributed in southern and coastal areas of China. The substitution rate of CTV was 4.70 × 10^− 4^ subs/site/year (95% credibility interval: 1.10 × 10^− 4^ subs/site/year ~ 9.10 × 10^− 4^ subs/site/year), with a slight increasing trend in CTV populations between 1990 and 2006. The CTV isolates in China shared a most common recent ancestor around 1875 (95% credibility interval: 1676.57 ~ 1961.02). The CTV in China was originated from wild citrus in Hunan and Jiangxi, and then spread from the wild citrus to cultivated citrus in the growing regions of Sichuan, Chongqing, Hubei, Fujian, Zhejiang, Guangxi and Guangdong provinces.

**Conclusions:**

This study has proved that CTV in China was originated from wild citrus in Hunan and Jiangxi. The spatial-temporal distribution and dispersal patterns has uncovered the population and pandemic history of CTV, providing hints toward a better understanding of the spread and origin of CTV in China.

**Supplementary Information:**

The online version contains supplementary material available at 10.1186/s12985-023-02190-0.

## Background

The genus Citrus and related genera (*Fortunella*, *Poncirus*, *Eremocitrus*, and *Microcitrus*) belong to Rutaceae family and subfamily Aurantioideae, which comprise some of the most widely cultivated fruit crops worldwide [1]. Citrus fruits are rich in various bioactives such as flavonoids, essential oils, carotenoids, limonoids, and synephrines [2, 3]. These natural nutrients have been reported effective in protecting human from various ailments, including cancer and inflammation, digestive, and cardiovascular diseases. In 2016, Chinese citrus cultivated acreage was 2.56 million hectares, accounting for 27.09% of the world citrus cultivation area. The Chinese citrus annual yield was 35.92 million tons which was 24.53% of the world citrus yield at the same period. Increasing by 27.68% from 2016, by 2022, Chinese citrus cultivated acreage was 45.531 million acres, 1.32 tons/acre for our citrus groves in 2022, orange production in 2022 reached 7.6 million metric tons (https://www.fas.usda.gov/data/china-citrus-annual-5). Meanwhile, there are abundant wild citrus resources in China, such as *Citrus mangshanensis*, *Citrus cavaleriei* and *Citrus medica et al.* [3, 4]. But there are still many factors that limit the growth of the citrus cultivation area and production, such as diseases, drought, frigidity, soil salinization, among which citrus tristeza caused by CTV presents as one of the most damaging disasters [5–7].

CTV is a member of the genus *Closterovirus* of the family *Closteroviridae*, consisting of single-stranded, (+)-sense RNA genome of approximately 19.3 kb in length [8]. The genome contains 12 open reading frames (ORFs) [9]. Of these, OFR 7 also known as *p25* gene is 672 bp in length, encoding 97% of the outer coat proteins. The *p25*, *p20* and *p23* together function as RNA silencing suppressors [10]. CTV is mainly transmitted by grafting and aphid vectors, and aphid vector is the most efficient vector of CTV [11]. One of the most common aphid vectors of CTV is the brown orange aphid (*Aphis citricidus*), which has the strongest transmission capacity and can effectively transmit most CTV strains. There are three main symptoms of citrus tristeza disease: quick decline (QD), stem pitting (SP), and seedling yellow (SY). The most common one in China is SP type. Symptoms of SP type include obvious dark brown depression in plant xylem [5]. Between 1956 and 2000, Spain lost more than 10 million citrus trees because of CTV [12]. During 1930–2019, CTV destroyed more than 100 million citrus plants in Brazil, Argentina, the United States, Spain, and Israel [13]. In the late 1980s, China restructured its citrus industry with an increased proportion of honey pomelo, sweet orange, and Shatangju mandarin cultivation, which increased the risk of CTV. Chen et al. [14] examined 1,439 samples from nine citrus producing areas in China and found that the incidence of CTV was 55.73%.

The molecular characteristics and pathogenesis of CTV as a worldwide disease has been extensively studied, but limited attention has been given to the evolutionary dynamics of this virus, especially regarding its evolutionary dynamics and dispersal patterns in China, studies of the evolutionary dynamics have been reported in rice stripe virus, tomato mosaic virus, infectious bursal disease viruses [15–17]. Silva et al. [18] used the *p25* gene to estimate the rate of evolution of CTV, and the substitution rate was 1.5 × 10^− 4^ subs/site/year (95% credible interval: 1.73 × 10^− 4^ subs/site/year − 3.16 × 10^− 4^subs/site/year), which proved that CTV was an exceptionally slowly evolving virus. Davino et al. [19] studied the genetic relationship among CTV strains from Sicily, Italy. The Bayesian phylogenetic analysis revealed that mild and severe CTV isolates belonging to five different lineages were introduced to Sicily in 2002, four lineages co-circulated in Eastern Sicily, one lineage (composed of mild isolates) spread to distant areas of Sicily. Benítez-Galeano et al. [20] used *p25* genes to infer the substitution rate and spread of CTV-*NC* genotypes. Their results showed that the substitution rate of the CTV-*NC* genotype was 1.19 × 10^− 3^ subs/site/year, the most common recent ancestor appeared in 1977. And the genotype of CTV-*NC* was originated from the United States. However, all of these studies mentioned above performed phylogenetic analysis of CTV globally and did not perform Bayesian analysis of CTV in China. Critically, CTV has been found on wild citrus in China [21, 22], but none of these studies explored whether CTV originated from cultivated or wild citrus. While the study on CTV of evolutionary dynamics and dispersal patterns will give a lot of hints for CTV prevention.

In this study, we collected the CTV-infected citrus samples from several citrus production regions from China, including Sichuan province, Hubei province, Chongqing province, Hunan province, Zhejiang province, Jiangxi province, Fujian province, Guangxi province and Guangdong province, and related publicly available data of CTV were studied in this article. The purpose of this research was (a) determining the distribution of CTV in China, (b) inferring the history of CTV transmission in China, and (c) inferring whether CTV originated from wild or cultivated citrus.

## Materials and methods

### Sample collection

**Fig. 1:**
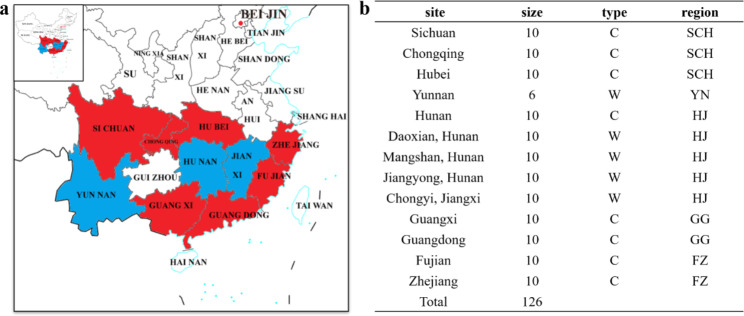
Sample site and sample collection. (**a**) Sample site in this study. Note: Colours indicate sample collection locations; red indicates the main wild citrus community and blue indicates the main cultivated citrus production area. (**b**) Sample number of each site in this study. Note: C: cultivated citrus; W: wild citrus; GG: Guangdong and Guangxi; FZ: Fujian and Zhejiang; HJ: Hunan and Jiangxi; SCH: Sichuan, Chongqing and Hubei; YN: Yunnan; HJ: Hunan and Jiangxi

For each sample, suspected CTV-infected leaves of four different directions (east, south, west, and north) from the same Newhall orange tree were cut off with a germ-free surgical scissors. The sampled leaves were placed in a Ziplock bag, transferred to the lab, and stored in the refrigerator at -80℃ until RNA extraction. The wild citrus samples were collected from Hunan and Jiangxi provinces, and the cultivated samples were collected from Sichuan, Chongqing, Hubei, Zhejiang, Fujian, Guangdong and Guangxi provinces. According to the different habitats and geographical regions of the citrus growing, the citrus producing area were divided into five groups as following (Fig. 1): SCH (Sichuan, Chongqing, and Hubei provinces), YN (Yunnan wild citrus main communities), HJ (Hunan cultivated citrus main producing areas, Hunan and Jiangxi wild citrus main communities), and GG (Guangxi and Guangdong provinces), and FZ group (Fujian and Zhejiang provinces; Fig. 1).

Besides, 39 CTV *p25* gene sequences and associated information including collection date and geographic location were retrieved from GenBank for this study. Those public data collected between 2005 and 2019 from citrus producing regions, including Hubei, Zhejiang, Hunan, Chongqing, Yunnan and Guangxi provinces (Supplementary Table 1).

### RNA extraction and p25 gene sequencing

Before extraction, the leaves stored under − 80℃ were grinded in liquid nitrogen. About 1 g of the grinded leaves for each sample was used for RNA extraction, following the instructions of TRIzol reagent (TaKaRa, Beijing, China). The RNA samples were stored under − 80℃ for the following steps.

Total RNA were reverse-transcribed using a PrimeScript™ RT reagent Kit with gDNA Eraser (Perfect Real Time, TaKaRa). CP gene was then amplified by regular PCR with the synthesized cDNA samples. Each PCR assay included the following components: 10× PCR buffer (Mg2 + plus; including 100 mM Tris-HCl, pH 8.9; 500 mM KCl; 15 mM MgCl2), 10 mM dNTP, 0.5 µM Forward primer, 0.5 µM Reverse primer, and 5 U/µl r-Taq, CP primers was designed by Michael *et al* [23] (CP-F: 5’-ATGGACGACGAAACAAAG-3’, CP-R:5’-TCAACGT GTGTTGAATTT-3’). Water was used as negative control, and cDNA of a CTV isolate was used as positive control. The PCR conditions included: one cycle at 94 ℃ for 5 min, then 35 cycles at 94 ℃ for 30 s, 55 ℃ for 30 s, and 72 ℃ for 45 s, with a final extension for 12 min at 72 ℃. The amplicons were visualized on 1.2% agarose gel using a gel imaging system (Universal Hood II, BIO-RAD, America) after electrophoresis for 30 min and stained with M5 Gel red dye. The positive cDNA samples that produced a correct amplicon were kept for further analysis and stored at -80℃. PCR products were ligated to PMD19-T vector (Tiangen, Beijing, China) and then subsequently propagated in E. coli strain DH5α. The recombinant plasmids were extracted and sequenced in both directions by Sangon Biological (Shanghai). At least four cDNA clones for each amplicon were sequenced to obtain a consensus sequence using SeqMan 7.1 software [24].

### Molecular variation analysis

To determine whether more pronounced genetic differences occurred between cultivated and wild citrus populations, we used the analysis of molecular variance (AMOVA) to partition the variation component among and within the population by GenALEx 6.502. Meanwhile, we computed the selective pressure with default arguments by MEGA-X, mainly obtained by calculating the replacement ratio of nonsynonymous codons and synonymous codons in the protein-coding sequence, namely, ω = d_N_/d_S_, where ω = 1 represents neutral mutation, ω < 1 means negative selection, and ω > 1 indicates positive selection [25].

### Phylogenetic analysis of CTV

To avoid bias in the phylogenetic, phylodynamic, and phylogeographic analysis, we used the RDP, GENECONV, MAXCHI, CHIMAERA, 3SEQ, BOOTSCAN, LARD, and SISCAN heuristic recombination detection methods implemented in the RDP 5.0 software [26] package with default settings (p-values less than 10^− 5^). Recombinats detected by at least four of the seven algorithms were considered to be true recombinats. Meanwhile, we use the Bayesian information criterion in ModelFinder [27] in IQ-TREE [28] software to select the substitution model.

To infer the substitution rate and timescale of CTV *p25* gene, we analyzed the *p25* gene sequences using a molecular clock. Firstly, we used Bayesian evaluation of temporal signal (BETS) [29] to test temporal structure in the data set. This method used the marginal likelihood (MLE) estimated by generalized stepping-stone sampling (GSS) to compare heterochronous model (*M*_het_) and isochronous model (*M*_iso_). Bayes factor (BF) was used to identify the best fit model to the data set, if log^(BF)^ = log^(P(Y|Mhet))^ – log^(P(Y|Miso))^ > 5 which indicate a strong preference of the *M*_het_ and providing decisive support for the presence of temporal signal in the data set [30, 31](Guy et al. 2016; Mathieu et al. 2020).

Using the substitution models, as selected above, the path sampling (PS) and stepping-stone sampling (SS) methods [32] were employed in BEAST v1.10 [33]. Two molecular clock models were combined (strict and uncorrelated lognormal relaxed) with three different demographic models (the constant-size coalescent, exponential growth coalescent, and Bayesian skyline coalescent) to choose the best combination model.

To infer the substitution rate and the most recent common ancestor of CTV in China, we used sampling time of each sequence to calibrate the molecular clock. Posterior distributions of parameters were estimated by Markov chain Monte Carlo (MCMC) sampling, and the best models were used to obtain maximum clade credibility (MCC) tree. All Bayesian analysis was run for 310 million steps with sampling every 31,000 steps, to ensure that the effective sampling size was greater than 200. After discarding the first 10% samples as burn-in, we used Tracer 1.71 to examined the convergence of MCMC chains (the effective sampling size greater than 200). Finally, the MCC tree was summarized using Tree Annotator v1.10.4 from the BEAST v1.10 package and visualized with FigTree 1.4.3.

### Phylogeographic and demographic history

To gain insight into the circulation of CTV across the citrus-producing of China, a phylogeographic analysis was conducted using Bayesian stochastic search variable selection (BSSVS) [34] to model the geographic transmission patterns in BEAST v1.10. Five geographic regions (SCH, YN, HJ, GG, and FZ regions) were coded as sampling location, and posterior distributions of parameters were estimated by MCMC sampling as described above. Besides, to test the robustness of the dispersal patterns of CTV migration in China, the dataset in this study were reanalysed by phylogeographic analysis with the data of YN merged into HJ group, which contains all the wild samples in this study. We run MCMC simulations for 100 million steps across three independent Markov chains and collected samples every 10,000 steps. After discarding the first 10% samples as burn-in, we used Tracer 1.71 to checked the effective sampling size, accepting only values higher than 200 for all the parameters.

The best-supported pair wise diffusions were identified using BF in SPREAD3 [35]. Migration pathways were considered to be of statistical significance when BF greater than 3 and mean posterior value greater than 0.5 [36]. We also estimated the number of expected location-state transitions (Markov jump counts) along the branches of the phylogeny [37].

## Results

### Sampling and CTV ***p25*** gene sequencing

Between 2018 and 2019, 524 leaf samples of suspected CTV were collected from wild and cultivated citrus in 10 provinces in China. Of these 524 samples, CTV was detected in 256 cultivated citrus samples and 136 wild citrus samples. Finally, CTV *p25* gene sequences were detected from 87 samples, including 47 cultivated citrus samples and 40 wild citrus samples (Fig. 1a). The obtained CTV *p25* gene sequences were deposited in the GenBank data bases under accession number OM371112-OM371198 (Supplementary Table 2). Meanwhile, 39 CTV *p25* gene sequences were downloaded from GenBank, all of which were from China and contained specific collection dates and locations. Notably, six of the 39 CTV *p25* gene sequences from GenBank were from wild citrus in YN, where the 87 sequences contributed by this study were from 10 different provinces and municipalities (Fig. 1b).

### The monophyly and temporal signal of the data set

None of the seven algorithms in the RDP package identified significant recombination signals (Supplementary Table 3). The detection of temporal signals shows that the *M*_het_ produces higher MLE values (-6223.19) relative to the *M*_iso_ (-6232.97). Both of these results indicated that our dataset could be used for the next phylogenetic analysis. IQ-Tree software showed that TN93 + F + I + G4 was the best substitution model, the BEAST v1.10 showed that the uncorrelated lognormal relaxed clock and the exponential growth model were the best fits to our sequence data, as these two models yielded higher log marginal likelihoods.

### Molecular variation and temporal dynamics of CTV

The AMOVA analysis (Supplementary Fig. 1) revealed that 47% of the genetic variation occurred between populations and 53% within populations. In addition, gene flow was a significant indicator of genetic variation among populations, and our calculations showed that CTV populations had less gene exchange (*N*_m_=0.557), indicating that CTV populations had genetic variation. The results of the selection pressure analysis showed that CTV populations were under negative selection (d_*N*_ = 0.01, d_*S*_ = 0.25, ω < 1).

The results of Bayesian phylogenetic analysis indicated that the CTV *p25* gene evolves at a rate of 4.70 × 10^− 4^ subs/site/year (95% credibility interval: 1.10 × 10^− 4^ subs/site/year to 9.10 × 10^− 4^ subs/site/year). With a most recent common ancestor in 1875 (95% credibility interval: 1676–1961), CTV was found to be present in HJ (root posterior probability = 0.94) at the first. In 1875, CTV diverged to form two lineages. Lineage 1 can be traced to HJ, diverged in 1923 (95% credibility interval: 1789–1979). Lineage 2 can be traced to YN, diverged in 1899 (95% credibility interval: 1716–1974). Interestingly, the source of all three geographic CTV populations was wild citrus.

**Fig. 2:**
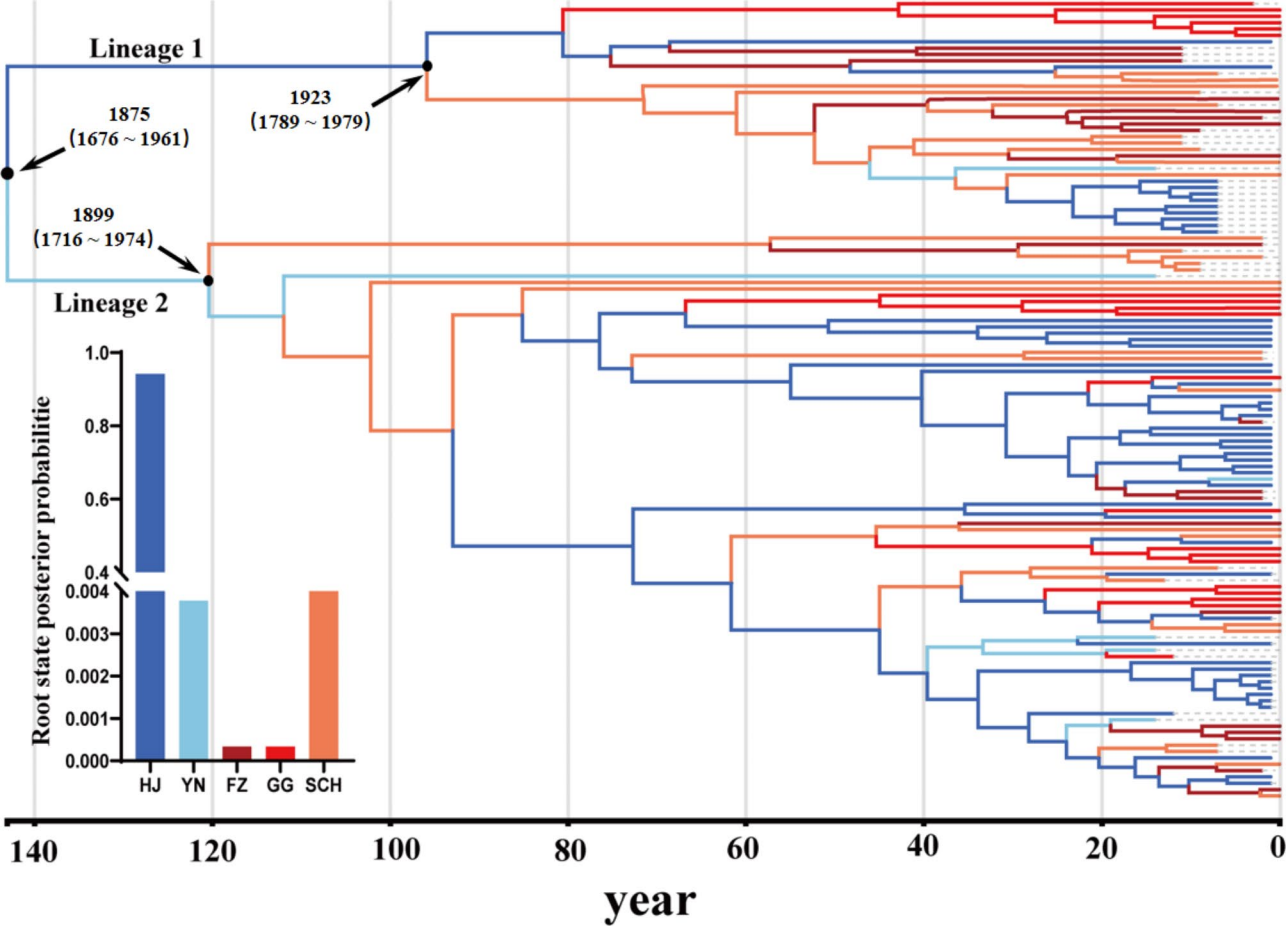
Time-scaled Bayesian MCC Phylogeny of CTV in China. Time-scaled maximum clade credibility tree inferred from *p25* sequences of CTV, total 126 sequences, samples were collected from GG, HJ, FZ, YN, SCH. Notes: Branch colours represent most probable inferred states, as indicated by the colour key. The inset panel was the root state posterior probability estimated for each region. GG: Guangdong, Guangxi provinces; HJ: Hunan, Jiangxi provinces; FZ: Fujian, Zhejiang provinces; YN: Yunnan province; SCH: Sichuan, Chongqing, Hubei provinces

### Dispersal spread trajectory and population dynamics of CTV

**Fig. 3:**
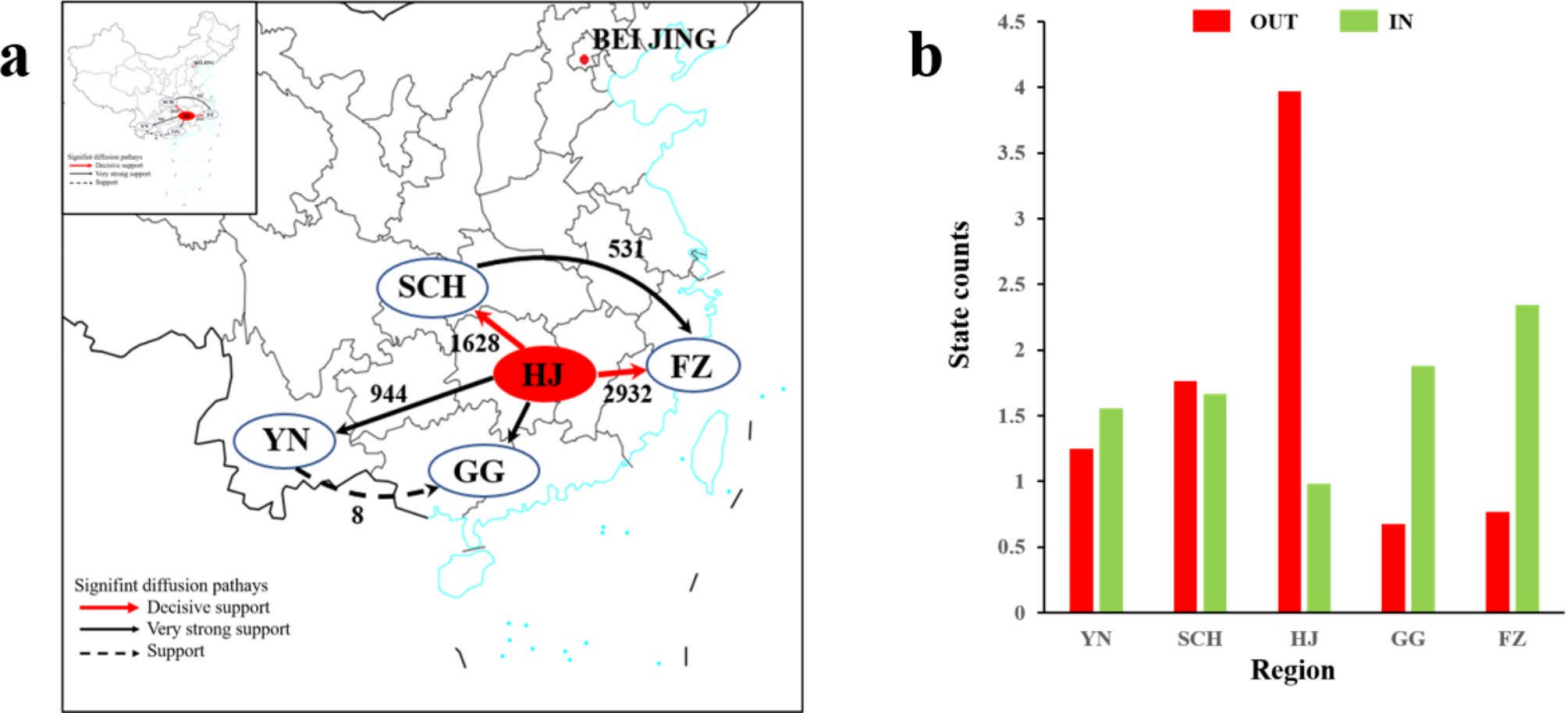
Spatial Diffusion of CTV in China. Spatial diffusion pathway (**a**) and histogram (**b**) of the total number of location state transitions inferred from 126 isolates collected from five citrus producing regions during 2005 and 2020. Note: Black numbers in (**a**) indicate BF values, red solid arrows: decisive support for BF > 1000, black solid arrows: very strong support for 150 < BF < 1000, black dashed arrows :3 < BF < 20; thickness indicates supported mobility. GG: Guangdong, Guangxi provinces; HJ: Hunan, Jiangxi provinces; FZ: Fujian, Zhejiang provinces; YN: Yunnan province; SCH: Sichuan, Chongqing, Hubei provinces

Bayesian phylogeographic analysis indicated the present of six CTV migration patterns (Fig. 3a), namely HJ to SCH (BF > 1000), HJ to FZ (BF > 1000), HJ to YN (150 < BF < 1000), HJ to GG (150 < BF < 1000), SCH to FZ (150 < BF < 1000) and YN to GG (3 < BF < 20). In 1857, CTV spread from HJ to YN, and in 1923, it spread toward north to SCH (Figs. 2 and 3a). The results were support by MCC tree (Fig. 2) and the diffusion spread trajectory map (Fig. 3b) with a much greater frequency of outbound export from HJ than from other regions. These results supported the present of four CTV migration links after data recombination (**Fig ****S1**** a**). Four CTV migration links were decisively supported: from YHJ to SCH (BF > 1000), from YHJ to GG (BF > 1000), from YHJ to FZ (BF > 1000), and from SCH to GG (BF > 1000). Meanwhile, these four CTV migration links had a common origin region – YHJ. This is further supported by the number of observed state changes (**Fig ****S1**** b**), with migration from YHJ being much greater than from any other regions.

**Fig. 4:**
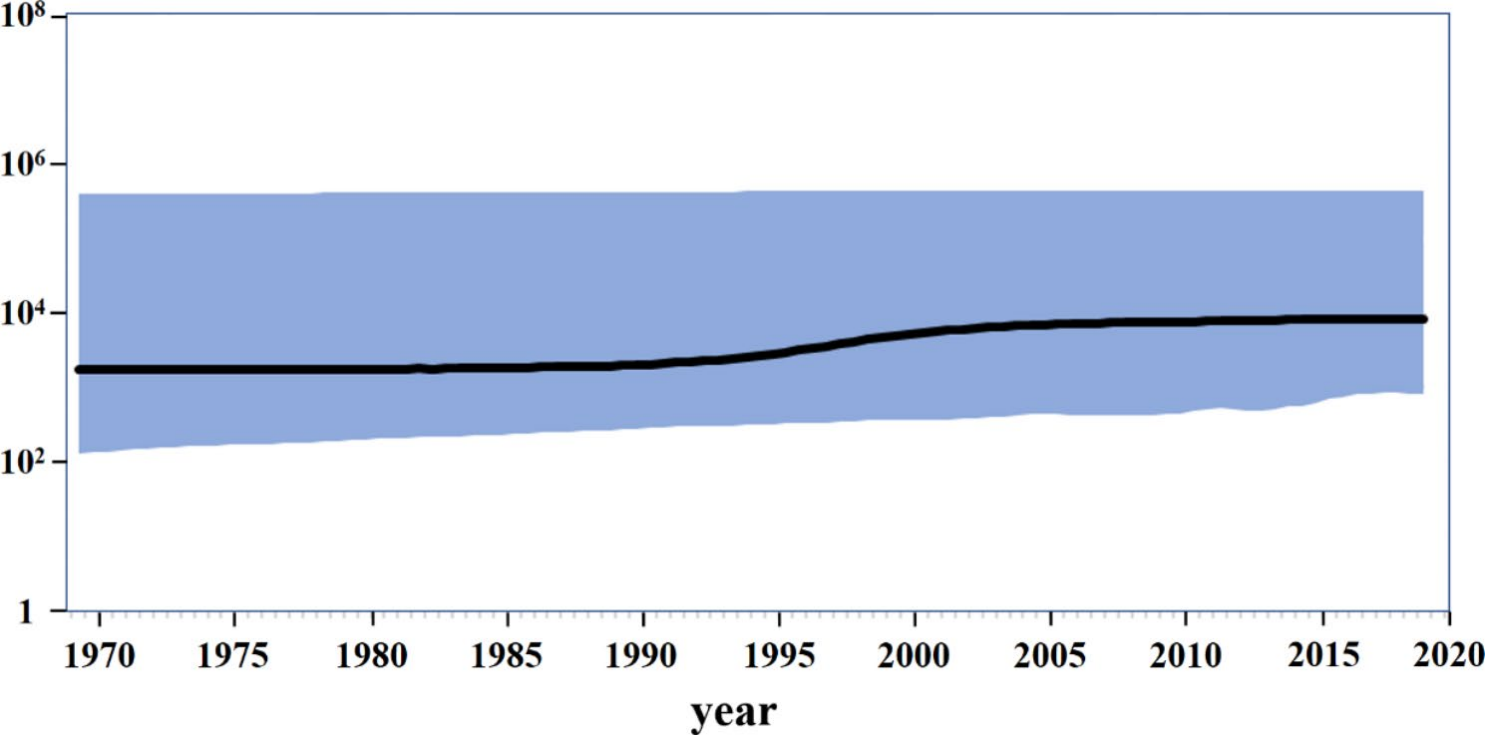
Population Dynamics of the CTV in China. Note: The y axis represented a measure of genetic diversity, given as the product of effective population size (Ne) and virus generation time (τ). The x axis was measured in calendar years. The black line showed the median estimate of the population size and the light blue shading showed the 95% credibility interval

Bayesian skyline plot revealed that the CTV population had experienced multiple shifts in history (Fig. 4). The population size of CTV *p25* gene in China remained stable until 1990. Between 1990 and 2006, the population size of CTV *p25* changed. The population of CTV *p25* remained stable after 2006. While, population size of CTV was higher than before.

## Discussion

In this study, we collected 126 CTV *p25* gene sequences for Bayesian phylogeographic analysis to infer the origin and transmission patterns of the CTV in China. The results showed that there were genetic differences among CTV in China (Fig. 2). CTV was found in all 10 provinces, while, from the overall trend (Fig. 1), CTV is mainly concentrated in the southern and coastal areas of which the geographical location near the Yangtze River Delta, Pearl River Delta, Hong Kong, Macao, Taiwan and Southeast Asia area. And these places located in temperate region. Possible reason to that would be the temperature and climate of these places are suitable for citrus planting, providing ideal host habitats for CTV and the aphids. Besides, these places are suitable for citrus import and export trade, and the accompanied with the import and export of citrus the spread of CTV happened. As reported Pakistan’s citrus exports increased every year (www.freshplaza.com), and CTV detection rates were also rising. The surveys revealed that the average incidence of CTV in 10 major citrus growing districts in the Northwest of Pakistan steadily increased from 24% (in 2002) to 31%, 35%, 39% and 44% in 2004, 2006, 2008, 2010, respectively [38]. These evidences indicate that the occurrence and spread of CTV are correlate with the citrus acreage in a degree.

The MCC tree (Fig. 2) showed that the most recent common ancestor of the CTV variant was in 1875 (95% confidence interval: 1676.57 ~ 1961.02), at the same period detected by molecular methods the first by Matsumoto et al. [39], and the substitution rate of CTV *p25* gene was 4.70 × 10^− 4^ subs/site/year (95% confidence interval: 1.10 × 10^− 4^ subs/site/year ~ 9.10 × 10^− 4^ subs/site/year). Silvan et al. [18] estimated the substitution rate of CTV *p25* gene was 1.58 × 10^− 4^ subs/site/year, and Benítez et al. [20] estimated the substitution rate of *NC* Lineage was 1.19 × 10^− 3^ subs/site/year. In this regard, it would be due to Benítez et al. inferred the substitution rate of *NC* Lineage only, whereas the study by Silvan et al., many strains were studied, and CTV is mostly mixed infection in the field. And the d_*N*_/d_*S*_ value of selective test in this study was 0.05, which was smaller than 1, indicating that CTV was under negative selection. In fact, as the main plant host of CTV, the citrus planting of China was changed in recent years. On the one hand, the large number of foreign introductions and domestic citrus varieties were planted (http://www.farmchina.org.cn/). As recorded, between 1972 and 2001, 356 citrus varieties were imported from abroad, of which 24 citrus varieties applied in production, like Newhall, Blood orange, Valencia late et al. On the other hand, more and more citrus varieties were crossed after those citruses were introduced, which made the varieties of citrus grown in China increasingly complex. Last decades has witnessed were rapid development of the Chinese citrus industry. Up to 2017, citrus planting acreage had reached 2.60 million hectares (https://www.fas.usda.gov/data/china-citrus-annual-3). With the growth of species and planting area, the positive rate of CTV increased and pesticide spraying was also increased. As a result, under those evolutionary pressures, such as hosts varieties, genotypes that are more adapted to environments would formatted either.

Along with the migration of citrus seedlings and artificial breeding, CTV dispersal breaks out. Phylogeographic analysis results that CTV originated from wild citrus in HJ, and then spread to cultivated citrus in the SCH, FZ and GG production areas and wild citrus in YN (Fig. 3a). The robustness of these patterns conformed by phylogeographic reanalysis as mentioned in the front section (Materials and Methods). CTV originated from wild citrus in YHJ, and then spread to cultivated citrus in the SCH, FZ and GG production areas (Supplementary Table 2), which emphasized the accuracy of the dispersal patterns of CTV, especially the spread of CTV from the wild to cultivated citrus in the SCH, FZ and GG production area. Divided into 3 stages (Fig. 4), the Bayesian skyline results presented the same clues. The first stage of Bayesian skyline was before 1990, the CTV population size basically remained stable. The second stage was between 1990 and 2005, of which the CTV population size showed an increasing trend. However, as reported that from December 1991 to spring 1992, most citrus production areas, the south of the Yangtze River in China, suffered from cold disasters (http://www.cma.gov.cn/). As a result, citrus planting area reduced sharply. While, the Bayesian skyline showed that CTV population size was not affected by the cold disasters (Fig. 4), and what’s interest was the population size got an expansion in the following 15 years. The consistent popular of CTV on the one hand, would be due to the host diversity, such as Passiflora [40] and so on. On the other hand, at the end of the 20th century and early 21th century (http://www.farmchina.org.cn/), the planting species and acreage in China has increased. These kinds of navel orange imported from abroad accompanied with increasing planting acreage, not only has offset the impact of freezing injury to citrus industry, but also has induced the expansion of the CTV population. The third stage was that after 2005, in this stage, more and more lines such as late maturing navel orange, Orah mandarin, Citrus reticulata ‘Ai Yuan 28’, Citrus reticulata ‘Bu ZhiHuo’ and so on developed rapidly.

## Conclusion

As the first attempt to monitor the CTV on a spatial-temporal scale in China, we have obtained a unique profile of the CTV distribution in China, and present a broad investigation of the CTV evolution. We answered several questions about CTV of China: first, the CTV in China was originated from wild citrus in HJ and then spread to the cultivated citrus; second, originated from wild, the CTV was then spread to wild citrus in YN, after that CTV spread to cultivated citrus throughout the country with YN and HJ as the center of dispersal (Figs. 2 and 3). The results of this study provided a reference for tracing the origin and spread of CTV in China, and providing a lot of hints for instructive advises to the prevention and control of CTV. A broad investigation of CTV evolution that samples collected from different host and even the word-wide should be conducted, in order to a better understanding of the mechanism of CTV origin, evolution and breakout would be developed and strategies can be proposed for prevention and control of CTV with high efficiency by low cost.

### Electronic supplementary material

Below is the link to the electronic supplementary material.

Supplementary Material 1

Supplementary Material 2

Supplementary Material 3

Supplementary Material 4

## Data Availability

All data applied for this study has been upload to Genbank, and the accession numbers were supplied in Supplementary Table 1.
