# Supplementary material for: Bayesian phylodynamic analysis reveals the evolutionary history and the dispersal patterns of citrus tristeza virus in China based on the p25 gene
Source: Virol J. 2023 Oct 3;20:223. doi: 10.1186/s12985-023-02190-0 (PMC10548698; doi:10.1186/s12985-023-02190-0)

**Supplementary Figure 1 Spatial diffusion of CTV in China.** Spatial diffusion pathway and histogram of the total number of location state transitions inferred from 126 isolates collected from four citrus-producing regions during 2005–2020. Note: “YHJ” indicates wild citrus production areas of Yunnan, Hunan, and Jiangxi.


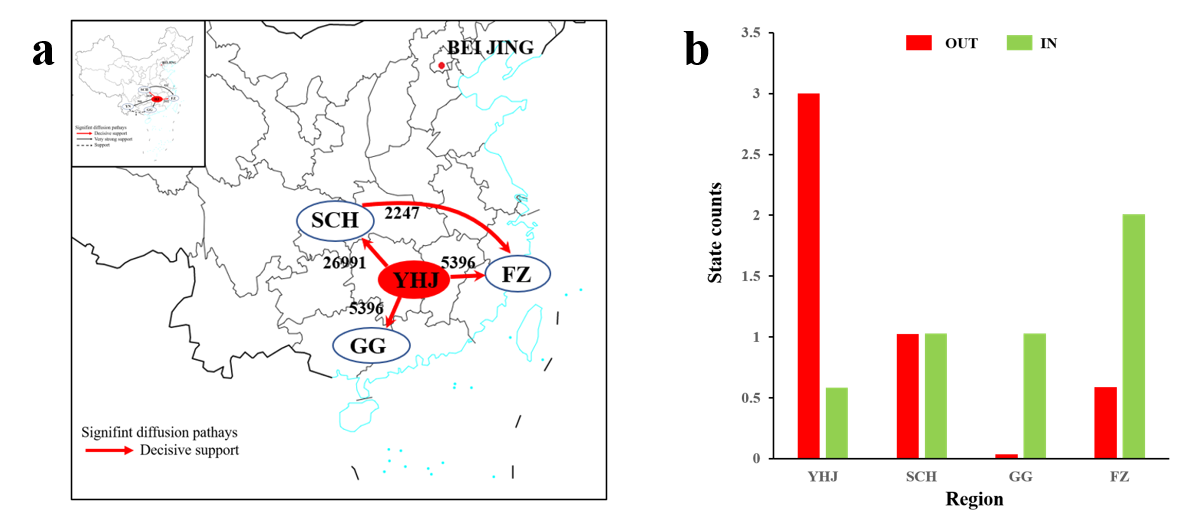

Supplement: Supplementary file 1 — Supplementary Material 1 [file 12985_2023_2190_MOESM1_ESM.docx]
