# Supplementary material for: Bayesian phylodynamic analysis reveals the evolutionary history and the dispersal patterns of citrus tristeza virus in China based on the p25 gene
Source: Virol J. 2023 Oct 3;20:223. doi: 10.1186/s12985-023-02190-0 (PMC10548698; doi:10.1186/s12985-023-02190-0)
Supplement: Supplementary file 2 — Supplementary Material 2 [file 12985_2023_2190_MOESM2_ESM.docx]

**Supplementary Table 1. AMOVA for all CTV p25 gene sequences.**

| Source of variation | d.f | Sum of squares | Variance components | Percentage of variation |
| --- | --- | --- | --- | --- |
| Among populations | 12 | 2405.592 | 18.568 | 47% |
| Within populations | 113 | 2336.567 | 20.678 | 53% |
| GeneFlow | *N*_m_: 0.557 |  |  |  |
