# Supplementary material for: Bayesian phylodynamic analysis reveals the evolutionary history and the dispersal patterns of citrus tristeza virus in China based on the p25 gene
Source: Virol J. 2023 Oct 3;20:223. doi: 10.1186/s12985-023-02190-0 (PMC10548698; doi:10.1186/s12985-023-02190-0)
Supplement: Supplementary file 3 — Supplementary Material 3 [file 12985_2023_2190_MOESM3_ESM.docx]

**Supplementary Table 3 Location and orgin of recomoination events present in wild and cultivated citrus of *citrus tristeza virus*.** Recombination events were identified based on analysis of 126 CTV sequences.

| **Event no.** | **Found in** | **Recomb.** | **Major parent** | **Minor parent** | **Detection methods** | | | | | | |
| --- | --- | --- | --- | --- | --- | --- | --- | --- | --- | --- | --- |
|  |  |  |  |  | **R** | **G** | **B** | **M** | **C** | **S** | **T** |
| 1 | 3 | CQ_CP_8_2019 | ZJ_FJ622883.1_2008 | Unknown | **-** | **-** | **-** | **+** | **-** | **-** | **+** |
| 2 | 1 | ZJ_FJ622884.1_2008 | ZJ_FJ622885.1_2008 | ZJ_FJ622883.1_2008 | **-** | **-** | **-** | **+** | **-** | **+** | **-** |
| 3 | 1 | CQ_MH323441.1_2017 | HB_FJ446482.1_2008 | SC_KF144759_2012 | **-** | **-** | **-** | **+** | **-** | **+** | **-** |
| 4 | 19 | MS_CP_8_2018 | Unknown | SC_KF144759_2012 | **-** | **-** | **-** | **-** | **-** | **+** | **+** |
| 5 | 1 | HB_KF144738_2010 | SC_KF144759_2012 | CQ_CP_7_2019 | **-** | **-** | **-** | **-** | **-** | **+** | **+** |

RDP (R), GENECONV (G), Bootscan (B), MaxChin (M), Chimaera (C), SiScan (S), 3Seq (T). “+” indicates detected recombination event, “-” indicates no recombination event detected.
