# Supplementary material for: Bayesian phylodynamic analysis reveals the evolutionary history and the dispersal patterns of citrus tristeza virus in China based on the p25 gene
Source: Virol J. 2023 Oct 3;20:223. doi: 10.1186/s12985-023-02190-0 (PMC10548698; doi:10.1186/s12985-023-02190-0)
Supplement: Supplementary file 4 — Supplementary Material 4 [file 12985_2023_2190_MOESM4_ESM.docx]

**Supplementary Table 2 Sample information of the samples collected in this study.** The *p25* gene sequence collection of Chinese *citrus tristeza virus*, including samples collected from wild and orchard.

| **Sample ID** | **Site** | **Type** | **Group** | **Data source** | **Collection time** | **Genbank** |
| --- | --- | --- | --- | --- | --- | --- |
| HU-PSTS | Hunan | C | HJ | public | 2007 | KU720382 |
| XT40-8 | Hunan | C | HJ | public | 2012 | KF144718 |
| XT40-2 | Hunan | C | HJ | public | 2012 | KF144719 |
| XT40-1 | Hunan | C | HJ | public | 2012 | KF144720 |
| XT38-8 | Hunan | C | HJ | public | 2012 | KF144721 |
| XT38-3 | Hunan | C | HJ | public | 2012 | KF144722 |
| XT37-7 | Hunan | C | HJ | public | 2012 | KF144724 |
| XT37-3 | Hunan | C | HJ | public | 2012 | KF144725 |
| XT34-7 | Hunan | C | HJ | public | 2012 | KF144726 |
| XT34-3 | Hunan | C | HJ | public | 2012 | KF144727 |
| DX_CP_1_2018 | Daoxian, Hunan | W | HJ | this study | 2018 | OM371169 |
| DX_CP_2_2018 | Daoxian, Hunan | W | HJ | this study | 2018 | OM371170 |
| DX_CP_3_2018 | Daoxian, Hunan | W | HJ | this study | 2018 | OM371171 |
| DX_CP_4_2018 | Daoxian, Hunan | W | HJ | this study | 2018 | OM371172 |
| DX_CP_5_2018 | Daoxian, Hunan | W | HJ | this study | 2018 | OM371173 |
| DX_CP_6_2018 | Daoxian, Hunan | W | HJ | this study | 2018 | OM371174 |
| DX_CP_7_2018 | Daoxian, Hunan | W | HJ | this study | 2018 | OM371175 |
| DX_CP_8_2018 | Daoxian, Hunan | W | HJ | this study | 2018 | OM371176 |
| DX_CP_9_2018 | Daoxian, Hunan | W | HJ | this study | 2018 | OM371177 |
| DX_CP_10_2018 | Daoxian, Hunan | W | HJ | this study | 2018 | OM371178 |
| MS_CP_1_2018 | Mangshan, Hunan | W | HJ | this study | 2018 | OM371179 |
| MS_CP_2_2018 | Mangshan, Hunan | W | HJ | this study | 2018 | OM371180 |
| MS_CP_3_2018 | Mangshan, Hunan | W | HJ | this study | 2018 | OM371181 |
| MS_CP_4_2018 | Mangshan, Hunan | W | HJ | this study | 2018 | OM371182 |
| MS_CP_5_2018 | Mangshan, Hunan | W | HJ | this study | 2018 | OM371183 |
| MS_CP_6_2018 | Mangshan, Hunan | W | HJ | this study | 2018 | OM371184 |
| MS_CP_7_2018 | Mangshan, Hunan | W | HJ | this study | 2018 | OM371185 |
| MS_CP_8_2018 | Mangshan, Hunan | W | HJ | this study | 2018 | OM371186 |
| MS_CP_9_2018 | Mangshan, Hunan | W | HJ | this study | 2018 | OM371187 |
| MS_CP_10_2018 | Mangshan, Hunan | W | HJ | this study | 2018 | OM371188 |
| JY_CP_1_2018 | Jiangyong, Hunan | W | HJ | this study | 2018 | OM371189 |
| JY_CP_2_2018 | Jiangyong, Hunan | W | HJ | this study | 2018 | OM371190 |
| JY_CP_3_2018 | Jiangyong, Hunan | W | HJ | this study | 2018 | OM371191 |
| JY_CP_4_2018 | Jiangyong, Hunan | W | HJ | this study | 2018 | OM371192 |
| JY_CP_5_2018 | Jiangyong, Hunan | W | HJ | this study | 2018 | OM371193 |
| JY_CP_6_2018 | Jiangyong, Hunan | W | HJ | this study | 2018 | OM371194 |
| JY_CP_7_2018 | Jiangyong, Hunan | W | HJ | this study | 2018 | OM371195 |
| JY_CP_8_2018 | Jiangyong, Hunan | W | HJ | this study | 2018 | OM371196 |
| JY_CP_9_2018 | Jiangyong, Hunan | W | HJ | this study | 2018 | OM371197 |
| JY_CP_10_2018 | Jiangyong, Hunan | W | HJ | this study | 2018 | OM371198 |
| CY_CP_1_2018 | Chongyi, Jiangxi | W | HJ | this study | 2018 | OM371159 |
| CY_CP_2_2018 | Chongyi, Jiangxi | W | HJ | this study | 2018 | OM371160 |
| CY_CP_3_2018 | Chongyi, Jiangxi | W | HJ | this study | 2018 | OM371161 |
| CY_CP_4_2018 | Chongyi, Jiangxi | W | HJ | this study | 2018 | OM371162 |
| CY_CP_5_2018 | Chongyi, Jiangxi | W | HJ | this study | 2018 | OM371163 |
| CY_CP_6_2018 | Chongyi, Jiangxi | W | HJ | this study | 2018 | OM371164 |
| CY_CP_7_2018 | Chongyi, Jiangxi | W | HJ | this study | 2018 | OM371165 |
| CY_CP_8_2018 | Chongyi, Jiangxi | W | HJ | this study | 2018 | OM371166 |
| CY_CP_9_2018 | Chongyi, Jiangxi | W | HJ | this study | 2018 | OM371167 |
| CY_CP_10_2018 | Chongyi, Jiangxi | W | HJ | this study | 2018 | OM371168 |
| CT11A | Chongqing | C | SCH | public | 2006 | JQ911664 |
| CN-L1-ZT1 | Chongqing | C | SCH | public | 2017 | MH323441 |
| CN-M1-ZT1 | Chongqing | C | SCH | public | 2017 | MH323442 |
| CQ_CP_1_2019 | Chongqing | C | SCH | this study | 2019 | OM371141 |
| CQ_CP_2_2019 | Chongqing | C | SCH | this study | 2019 | OM371142 |
| CQ_CP_3_2019 | Chongqing | C | SCH | this study | 2019 | OM371143 |
| CQ_CP_4_2019 | Chongqing | C | SCH | this study | 2019 | OM371144 |
| CQ_CP_5_2019 | Chongqing | C | SCH | this study | 2019 | OM371145 |
| CQ_CP_6_2019 | Chongqing | C | SCH | this study | 2019 | OM371146 |
| CQ_CP_7_2019 | Chongqing | C | SCH | this study | 2019 | OM371147 |
| Y8 | Hubei | C | SCH | public | 2008 | FJ446482 |
| Y5 | Hubei | C | SCH | public | 2008 | FJ446483 |
| G5 | Hubei | C | SCH | public | 2008 | FJ446484 |
| S45-7 | Hubei | C | SCH | public | 2010 | KF144738 |
| S45-3 | Hubei | C | SCH | public | 2010 | KF144739 |
| S39-7 | Hubei | C | SCH | public | 2010 | KF144741 |
| S39-6 | Hubei | C | SCH | public | 2010 | KF144742 |
| HB_CP_1_2019 | Hubei | C | SCH | this study | 2019 | OM371112 |
| HB_CP_2_2019 | Hubei | C | SCH | this study | 2019 | OM371113 |
| HB_CP_3_2019 | Hubei | C | SCH | this study | 2019 | OM371114 |
| SC-BSH-1 | Sichuan | C | SCH | public | 2012 | KF144755 |
| SC-BSH-5 | Sichuan | C | SCH | public | 2012 | KF144757 |
| SC-PG-4 | Sichuan | C | SCH | public | 2012 | KF144758 |
| SC-WZ-6 | Sichuan | C | SCH | public | 2012 | KF144759 |
| SC-Z-1-1-10 | Sichuan | C | SCH | public | 2012 | KF144761 |
| CN-RB-9 | Sichuan | C | SCH | public | 2017 | MH558665 |
| CN-RB-L13 | Sichuan | C | SCH | public | 2017 | MH558666 |
| SC_CP_1_2019 | Sichuan | C | SCH | this study | 2019 | OM371148 |
| SC_CP_2_2019 | Sichuan | C | SCH | this study | 2019 | OM371149 |
| SC_CP_3_2019 | Sichuan | C | SCH | this study | 2019 | OM371150 |
| CT-W1 | Yunnan | W | YN | public | 2005 | FJ998191 |
| CT-W2 | Yunnan | W | YN | public | 2005 | FJ998192 |
| CT-W3 | Yunnan | W | YN | public | 2005 | FJ998193 |
| CT-W4 | Yunnan | W | YN | public | 2005 | FJ998194 |
| CT-W5 | Yunnan | W | YN | public | 2005 | FJ998195 |
| YN1-6 | Yunnan | W | YN | public | 2018 | MW365403 |
| GD_CP_1_2019 | Guangdong | C | GG | this study | 2019 | OM371125 |
| GD_CP_2_2019 | Guangdong | C | GG | this study | 2019 | OM371126 |
| GD_CP_3_2019 | Guangdong | C | GG | this study | 2019 | OM371127 |
| GD_CP_4_2019 | Guangdong | C | GG | this study | 2019 | OM371128 |
| GD_CP_5_2019 | Guangdong | C | GG | this study | 2019 | OM371129 |
| GD_CP_6_2019 | Guangdong | C | GG | this study | 2019 | OM371130 |
| GD_CP_7_2019 | Guangdong | C | GG | this study | 2019 | OM371131 |
| GD_CP_8_2019 | Guangdong | C | GG | this study | 2019 | OM371132 |
| GD_CP_9_2019 | Guangdong | C | GG | this study | 2019 | OM371133 |
| GD_CP_10_2019 | Guangdong | C | GG | this study | 2019 | OM371134 |
| GX_CP_1_2019 | Guangxi | C | GG | this study | 2019 | OM371156 |
| GX_CP_2_2019 | Guangxi | C | GG | this study | 2019 | OM371157 |
| GX_CP_3_2019 | Guangxi | C | GG | this study | 2019 | OM371158 |
| GX_CP_4_2019 | Guangxi | C | GG | this study | 2019 | OM371151 |
| GX_CP_5_2019 | Guangxi | C | GG | this study | 2019 | OM371152 |
| GX_CP_6_2019 | Guangxi | C | GG | this study | 2019 | OM371153 |
| GX_CP_7_2019 | Guangxi | C | GG | this study | 2019 | OM371154 |
| GX_CP_8_2019 | Guangxi | C | GG | this study | 2019 | OM371155 |
| CT-W11 | Guangxi | C | GG | public | 2007 | FJ998201 |
| CT91-A1 | Guangxi | C | GG | public | 2016 | MH593380 |
| FJ_CP_1_2019 | Fujian | C | FZ | this study | 2019 | OM371115 |
| FJ_CP_2_2019 | Fujian | C | FZ | this study | 2019 | OM371116 |
| FJ_CP_3_2019 | Fujian | C | FZ | this study | 2019 | OM371117 |
| FJ_CP_4_2019 | Fujian | C | FZ | this study | 2019 | OM371118 |
| FJ_CP_5_2019 | Fujian | C | FZ | this study | 2019 | OM371119 |
| FJ_CP_6_2019 | Fujian | C | FZ | this study | 2019 | OM371120 |
| FJ_CP_7_2019 | Fujian | C | FZ | this study | 2019 | OM371121 |
| FJ_CP_8_2019 | Fujian | C | FZ | this study | 2019 | OM371122 |
| FJ_CP_9_2019 | Fujian | C | FZ | this study | 2019 | OM371123 |
| FJ_CP_10_2019 | Fujian | C | FZ | this study | 2019 | OM371124 |
| Z14 | Zhejiang | C | FZ | public | 2008 | FJ622884 |
| CS-7 | Zhejiang | C | FZ | public | 2010 | HQ634290 |
| ZJ_CP_1_2017 | Zhejiang | C | FZ | this study | 2017 | OM371135 |
| ZJ_CP_2_2017 | Zhejiang | C | FZ | this study | 2017 | OM371136 |
| ZJ_CP_3_2017 | Zhejiang | C | FZ | this study | 2017 | OM371137 |
| ZJ_CP_4_2017 | Zhejiang | C | FZ | this study | 2017 | OM371138 |
| ZJ_CP_5_2017 | Zhejiang | C | FZ | this study | 2017 | OM371139 |
| ZJ_CP_6_2017 | Zhejiang | C | FZ | this study | 2017 | OM371140 |
| Z24 | Zhejiang | C | FZ | public | 2008 | FJ622885 |
| Z8 | Zhejiang | C | FZ | public | 2008 | FJ622883 |
